# Seroprevalence of *Vibrio cholerae* in Adults, Haiti, 2017

**DOI:** 10.3201/eid2909.230401

**Published:** 2023-09

**Authors:** Wilfredo R. Matias, Yodeline Guillaume, Gertrude Cene Augustin, Kenia Vissieres, Ralph Ternier, Richelle C. Charles, Jason B. Harris, Molly F. Franke, Louise C. Ivers

**Affiliations:** Brigham and Women’s Hospital Division of Infectious Diseases, Boston, Massachusetts, USA (W.R. Matias);; Massachusetts General Hospital Center for Global Health, Boston (W.R. Matias, Y. Guillaume);; Massachusetts General Hospital Division of Infectious Diseases, Boston (W.R. Matias, R.C. Charles, J.B. Harris, L.C. Ivers); Zanmi Lasante, Croix-des-Bouquets, Haiti (G. Cene Augustin, K. Vissieres, R. Ternier);; Harvard Medical School, Boston (R.C. Charles, J.B. Harris, M.F. Franke, L.C. Ivers);; Harvard School of Public Health, Boston (R.C. Charles);; Harvard Global Health Institute, Cambridge, Massachusetts, USA (L.C. Ivers)

**Keywords:** cholera, Haiti, *Vibrio cholerae*, seroepidemiology, antibodies, dried blood spots, viruses, vaccine-preventable diseases

## Abstract

In Haiti in 2017, the prevalence of serum vibriocidal antibody titers against *Vibrio cholerae* serogroup O1 among adults was 12.4% in Cerca-la-Source and 9.54% in Mirebalais, suggesting a high recent prevalence of infection. Improved surveillance programs to monitor cholera and guide public health interventions in Haiti are necessary.

In 2010, cholera, caused by the bacterium *Vibrio cholerae*, was introduced into Haiti, resulting in >800,000 cases and >10,000 deaths (*1*,*2*). Case incidence peaked in 2012, then decreased, and the last case of confirmed cholera was reported in February 2019 (*3*). More than 3 years later, in October 2022, cholera was again detected in Haiti, and that outbreak is ongoing (*4*,*5*).

The response to cholera in Haiti and globally has been hampered by inaccuracies in estimating the actual prevalence of disease (*6*). In resource-limited settings where infectious diseases surveillance systems and laboratory capacity are limited, clinical case count–guided public health interventions can be suboptimal because of limitations in the accuracy of clinical case definitions (*7*). More accurate estimates of cholera disease prevalence and transmission dynamics are key for guiding and monitoring control efforts. Serosurveillance represents a promising tool to address the limitations of clinical surveillance (*8*,*9*). However, seroepidemiologic data are lacking from settings like Haiti where cholera has resurged. In 2017, we conducted a seroepidemiologic survey to measure the prevalence of cholera in Haiti during the waning phase of the first cholera epidemic in that country.

## The Study

This study was conducted as part of a campaign to control and eliminate cholera transmission in 2 communities in Haiti. The first, Cerca-la-Source, is a rural, mountainous community of ≈50,000 persons. The second, Mirebalais, is an urban commune of ≈100,000 persons. Both communities are located in the Centre Department of Haiti, a historically underserved and particularly impoverished region of the country. 

We conducted a census of both communities. During the census, a subset of households was invited to participate in a household survey and a serologic survey at fixed sampling intervals during March–August 2017 (Appendix Table 1). Trained study enumerators implemented study procedures in their native language of Haitian Creole; the procedures included surveys to measure self-reported sociodemographic and cholera risk factors.

**Table 1:** Unweighted demographic characteristics of *Vibrio cholerae* serosurvey participants compared with census participants in 2 communities, Centre Department, Haiti, March–August 2017*

Characteristic	Cerca-la-Source				Mirebalais		
	Census†	Serosurvey			Census†		Serosurvey
Total no.	24,500		156		45,365		121
Sex							
M	12,157 (49.6)		74 (47.4)		21,397 (47.2)		56 (46.3)
F	12,343 (50.4)		82 (52.6)		23,968 (52.8)		65 (53.7)
Mean age (SD)	37.1 (16.4)		42.3 (16.1)		37.1 (16.3)		44.6 (16.8)
Age group, y							
18–30	11,162 (45.6)		47 (30.1)		20,700 (45.6)		31 (25.6)
31–40	4,899 (20.0)		29 (18.6)		9,214 (20.3)		25 (20.7)
41–50	3,728 (15.2)		32 (20.5)		6,519 (14.4)		25 (20.7)
>50	4,711 (19.2)		48 (30.8)		8,932 (19.7)		40 (33.1)
Communal section							
1st Acajou Bruler	9,298 (38.0)	51 (32.7)			NA	NA	
2nd Acajou Bruler	7,952 (32.5)	59 (37.8)			NA	NA	
3rd Lamielle (Cerca-la-Source)	7,250 (29.6)	46 (29.5)			NA	NA	
3rd Grand Boucan	NA	NA			26,202 (57.8)	67 (55.4)	
6th Sarazin	NA	NA			19,163 (42.2)	54 (44.6)	

*Values are no. (%) except as indicated. NA, data not applicable for this category. †Census only includes persons >18 years of age because that was the population included in the serosurvey.

We obtained dried blood spots from consenting adults >18 years of age and shipped them to a laboratory in Boston, Massachusetts, USA, where we performed vibriocidal assays by using a drop-plate method from dried blood spots specimens, as described previously (*10*), except we used Advance Dx100 Serum Separator cards (Advance Dx, Inc., https://adx100.com) instead of the cards used in that study. We used target *V. cholerae* strains 19479 El Tor Inaba and X25049 El Tor Ogawa.

To ensure estimates were representative of the populations of Mirebalais and Cerca-la-Source, we used a raking procedure to apply survey weights on the basis of the population distribution of age, sex, and communal sections from the census in those regions. We used a random intercept to account for clustering by household. The primary outcome was the overall seroprevalence (either Ogawa or Inaba) of vibriocidal antibody responses against *V. cholerae* for each community. We defined seropositivity as a vibriocidal antibody response titer threshold of >320 on the basis of the best available evidence, a recent study in Bangladesh that estimated that a vibriocidal modal titer of 320 had a sensitivity of 80.6% and specificity of 83.0% for infections within the preceding year (*9*). We also calculated serotype-specific seroprevalence estimates for each region. For potential risk factors for seropositivity, we provided descriptive statistics, weighted seroprevalence estimates, and 95% CIs (for categorical variables) and odds ratios (ORs) with 95% CIs. We calculated ORs by using univariable logistic regression followed by multivariable logistic regression analysis, including only those risk factors associated with cholera at a significance level of p<0.20 in univariable analysis. We conducted analyses using the survey package in R 4.2.2 (The R Project for Statistical Computing, https://cran.r-project.org) (*11*).

The study was approved by the Partners Healthcare Institutional Review Board (protocol 2016P002781) and the Zanmi Lasante Institutional Review Board (protocol ZL IRB ID AK). All study participants provided written informed consent.

Overall, we enrolled 265 (27.6%) of 960 invited households in the study. Samples from 48 households were lost during tumultuous sociopolitical events, resulting in samples from 217 households available for analysis: 99 households with 156 persons in Cerca-la-Source and 118 households with 121 persons in Mirebalais.

We analyzed unweighted demographic characteristics for the census population and for serosurvey participants (Table 1). Serosurvey participants were representative of the census population. The weighted seroprevalence of *V. cholerae* was 12.4% (95% CI 6.76%–20.0%) in Cerca-la-Source and 9.54% (95% CI 4.91%–16.0%) in Mirebalais (Table 2). We analyzed the frequency distribution of vibriocidal antibody titers for both serotypes (Figure). Only 4 of 277 persons reported having received oral cholera vaccine, consistent with the fact that no major public health oral cholera vaccine campaign had been undertaken in those regions before sample collection.

**Table 2:** Weighted seroprevalence based on vibriocidal antibody titers in *Vibrio cholerae* serosurvey participants in 2 communities, Centre Department, Haiti, March–August 2017*

Strain	Cerca-la-Source				Mirebalais		
	No. tested	No. positive	% Seroprevalence (95% CI)		No. tested	No. positive	% Seroprevalence (95% CI)
Either Ogawa or Inaba	156	16	12.4 (6.76–20.0)		121	12	9.54 (4.91–16.0)
Ogawa only	156	14	9.73 (5.38–16.0)		121	11	8.75 (4.28–15.0)
Inaba only	156	2	2.69 (0.49–8.00)		121	3	2.73 (0.57–7.00)

*Based on a vibriocidal antibody assay positivity threshold titer of 320. Weights were computed as the inverse probability of selection and adjusted so that the marginal distribution of age group, sex, and communal section agreed with those from census estimates.

**Figure:**
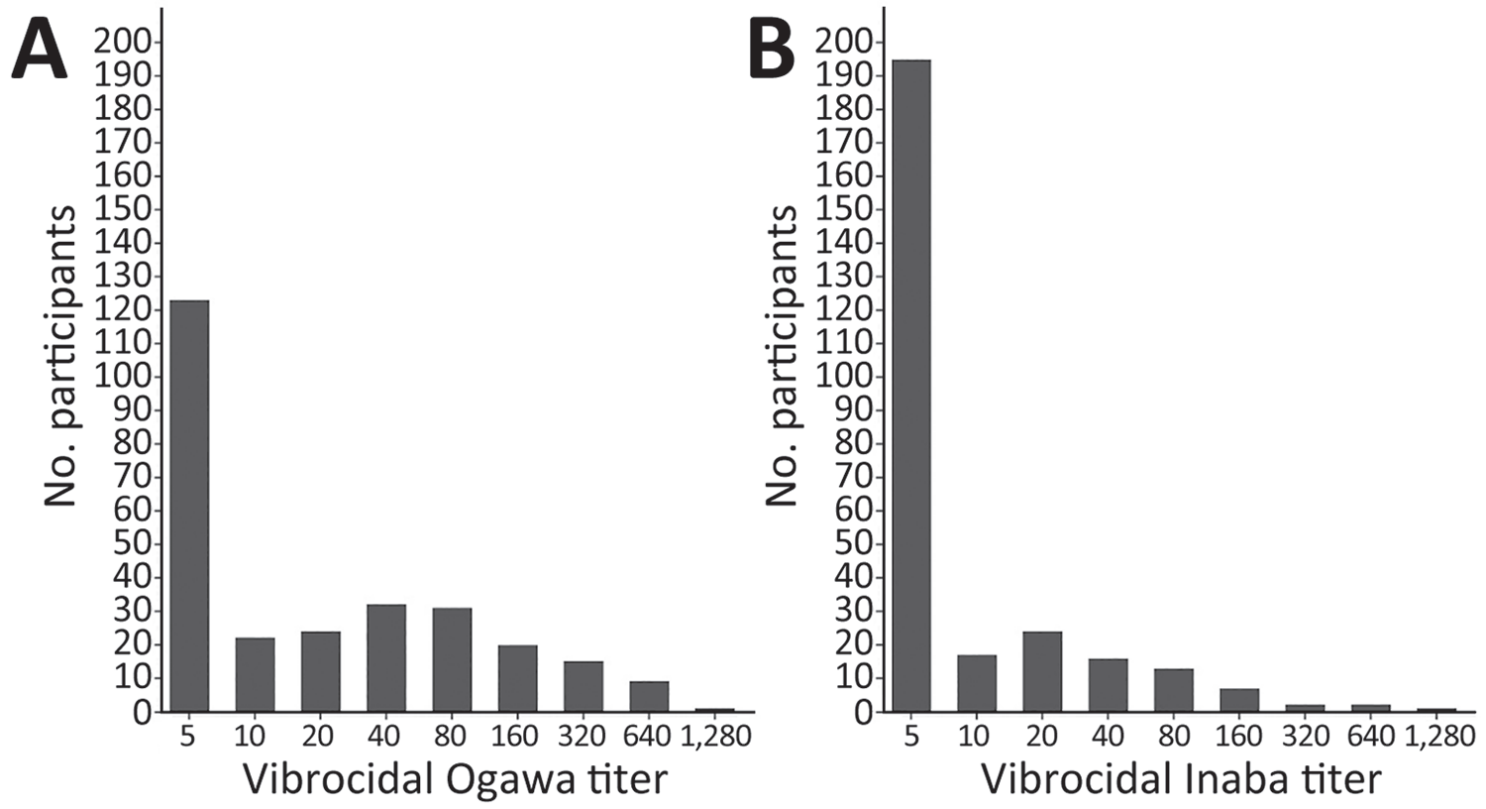
Serosurvey participants with vibriocidal antibody titers for Ogawa (A) and Inaba (B) *Vibrio cholerae* serotypes in 2 communities, Centre Department, Haiti, March–August 2017. Samples came from 217 total households, 99 (156 persons) in Cerca-la-Source and 118 (121 persons) in Mirebalais. All participants were adults >18 years of age.

We calculated seroprevalence estimates for potential risk factors for cholera (Appendix Table 2). Seropositivity varied across multiple subgroups; however, 95% CIs were wide. Only the poverty likelihood index (OR 2.33, 95% CI 0.93–5.84) and reporting having an unimproved toilet compared with open defecation (OR 0.26, 95% CI 0.04–1.53) met our predetermined p value threshold for inclusion into a multivariable model, so we did not perform multivariable analysis.

## Conclusions

The vibriocidal antibody is a complement-dependent, bactericidal antibody directed against the lipopolysaccharide O-antigen of *V. cholerae* and is the best characterized immunologic marker of recent exposure to cholera. However, there is no widely agreed-upon threshold to quantify exposure over a given period, and our understanding of the relationship between symptom severity and antibody kinetics is limited. In the study from Bangladesh, a vibriocidal titer of >320 was the best marker of infection in the preceding year (*9*).

Limited serologic data on *V. cholerae* are available from Haiti. One prior serosurvey, conducted during March–April 2011, within the first 6 months of the onset of the epidemic in the Artibonite Department of Haiti, estimated that 39% of persons had a vibriocidal titer >320, whereas 64% had titers >80, which suggested extensive infection and was consistent with high early case counts (*12*).

The findings from this study should be interpreted considering several limitations. Only adults >18 years of age in 1 department of Haiti participated, so the data cannot be directly extrapolated to younger age groups and other regions; however; during 2017–2018, that department was the most affected according to case counts (*13*). We were unable to account for uncertainty in vibriocidal assay performance characteristics. Ideally, seroprevalence estimates should integrate data on the local sensitivity and specificity of a serologic assay, which are not available for Haiti (*14*). Last, the survey was cross-sectional and did not account for temporal waning of serologic markers.

In summary, in 2017, the seroprevalence of *V. cholerae* vibriocidal antibodies was 12.4% in Cerca-la-Source and 9.54% in Mirebalais in Haiti, suggesting a high rate of recent infection even at a time when case incidence was declining. Although commune-level incidence data were not available for direct comparison, in 2017, the reported annual incidence for the Centre Department, where Cerca-la-Source and Mirebalais are located, was 4.3 cases/1,000 inhabitants, which offers a general frame of reference (*13*,*15*). Those findings inform our understanding of cholera epidemic dynamics in Haiti, which is now experiencing a resurgence of cholera after nearly 3 years without a confirmed case. Our results demonstrate a higher-than-expected disease prevalence and suggest the need for improved surveillance to monitor cholera and guide public health interventions, especially during the waning phase of outbreaks.

Appendix Additional information about seroprevalence of *Vibrio cholerae* in adults, Haiti, 2017.
