# Supplementary material for: Seroprevalence of Vibrio cholerae in Adults, Haiti, 2017
Source: Emerg Infect Dis. 2023 Sep;29(9):1929–32. doi: 10.3201/eid2909.230401 (PMC10461664; doi:10.3201/eid2909.230401)
Supplement: Appendix — Additional information about seroprevalence of Vibrio cholerae in adults, Haiti, 2017. [file 23-0401-Techapp-s1.pdf]

Article DOI: https://doi.org/10.3201/eid2909.230401

Seroprevalence of *Vibrio cholerae* in Adults, Haiti, 2017

Appendix

Appendix Table 1. Study population, date, and sampling intervals (*1*)

Characteristic	Cerca-la-Source	Mirebalais
Census – number of households	9,497	23,194
Census – number of individuals	48,799	97,755
Date of census and household survey	April 6 – May 23, 2017	March 24 – June 9, 2017
Interval for inclusion into household survey	Every 18th household in the census	Every 11th household in the census
Date of serosurvey	August 8 – August 17, 2017	May 22 – June 16, 2017
Interval for inclusion into serosurvey	Every 3rd household in household survey	Every 2nd household in household survey

Appendix Table 2. Seroprevalence by household-level risk factors and associations with seropositivity[a]

Characteristics (N, if not 277)	Total (N = 277)	Unweighted Seropositive (N = 28)	Unweighted Seronegative (N = 249)	Weighted Seroprevalence % (95% CI)	Weighted Odds Ratio (95% CI)	p-value
Sociodemographic characteristics						
Age						
18 - 30	78	10 (12.8)	68	10.7 (4.96 - 19.1)	Ref	Ref
31 - 40	54	7	47	15.9 (6.83 - 29.1)	1.58 (0.55 - 4.57)	0.4
41 - 50	57	4	53	5.94 (1.44 - 15.0)	0.53 (0.13 - 2.08)	0.36
>50	88	7	81	8.24 (3.14 - 16.6)	0.75 (0.24 - 2.31)	0.62
Gender						
Man	130	10	120	8.17 (3.98 - 14.3)	Ref	Ref
Woman	147	18	129	12.7 (7.15 - 20.3)	1.64 (0.68 - 3.94)	0.27
No. people living in household[b, c]		5 (3 - 7)	4 (3 - 6)		1.09 (0.89 - 1.34)	0.39
Children under 5 in household[b]		0 (0 - 2)	0 (0 - 1)		1.30 (0.75 - 2.25)	0.34
Likelihood of Poverty[b,] (*2*)		10.4 (2.2 - 54)	10.4 (2.2 - 35.6)		1.01 (0.99 - 1.03)	0.24
Likelihood of Poverty (Percentile)						
0 – 49	201	17	184	8.85 (4.96 – 14.2)	Ref	Ref
50 – 100	76	11	65	18.5 (9.18 – 31.1)	2.33 (0.93 – 5.84)	0.07
Household Hunger Scale (*3*)						
Little to no hunger	116	12	104	11.6 (5.80 - 19.8)	Ref	Ref
Moderate hunger	113	9	104	6.44 (2.78 - 12.2)	0.53 (0.19 - 1.45)	0.22
Severe hunger	48	7	41	18.1 (6.80 - 35.1)	1.68 (0.53 - 5.36)	0.38
Water and Hygiene						
Water source						
Unimproved	80	8	72	9.25 (3.57 - 18.4)	Ref	Ref
Improved	197	20	177	10.9 (6.51 - 16.6)	1.19 (0.04 - 1.20)	0.73

Characteristics (N, if not 277)	Total (N = 277)	Unweighted Seropositive (N = 28)	Unweighted Seronegative (N = 249)	Weighted Seroprevalence % (95% CI)	Weighted Odds Ratio (95% CI)	p-value
Time to retrieve water and return to house on foot (minutes)						
<15	167	15	152	9.23 (4.93 - 15.2)	Ref	Ref
> = 15 < = 30	71	10	61	15.1 (6.58 - 27.7)	1.75 90.63 - 4.90)	0.28
>30 to < = 60	30	2	28	4.86 (0.64 - 15.5)	0.5 (0.10 - 2.42)	0.39
>60	9	1	8	12.9 (0.14 - 62.6)	1.45 (0.19 - 11.0)	0.72
Money spent on water daily for household						
0 HTG[d]	198	19	179	9.56 (5.39 - 15.2)	Ref	Ref
Less than 15 HTG	25	1	24	6.28 (0.26 - 26.7)	0.63 (0.08 - 5.08)	0.67
15 - 30 HTG	34	4	30	11.7 (3.55 - 25.9)	1.25 (0.39 - 4.08)	0.71
More than 30 HTG	20	4	16	18.6 (4.01 - 44.7)	2.17 (0.53 - 8.79)	0.28
Water from the water source unavailable for at least 1 whole day in last 1 month						
No	123	12	111	8.56 (3.96 - 15.5)	Ref	Ref
Yes	154	16	138	11.9 (6.67 - 18.9)	1.44 (0.56 - 3.68)	0.44
Frequency of treating water						
Not always	135	9	126	7.87 (3.56 - 14.4)	Ref	Ref
Always or almost always	142	19	123	13.5 (7.59 - 21.4)	1.83 (0.70 - 4.76)	0.22
Household exposures						
Respondent had diarrhea in the last 2 weeks						
No	227	24	203	10.0 (6.19 - 15.0)	Ref	Ref
Yes	50	4	46	13.3 (3.67 - 30.4)	1.38 (0.40 - 4.73)	0.61
Respondent had diarrhea requiring stay overnight in a cholera treatment unit or hospital since 2010?						
No	228	23	205	10.6 (6.48 - 16.0)	Ref	Ref
Yes	49	5	44	10.3 (2.90 - 23.5)	0.96 (0.29 - 3.20)	0.95
Anyone in your household (besides respondent) had diarrhea in the last two weeks? (N = 271)						
No	197	18	179	9.89 (5.57 - 15.8)	Ref	Ref
Yes	74	10	64	13.7 (6.18 - 24.6)	1.44 (0.55 - 3.77)	0.45
Anyone in household (besides respondent) been diagnosed with cholera by a doctor in the last two weeks? (If diarrhea in last 2 weeks = Yes. N = 77)						
No	60	6	54	10.5 (3.52 - 22.2)	Ref	Ref
Yes	17	3	14	15.1 (2.28 - 41.7)	1.53 (0.27 - 8.72)	0.63
Anyone in your household (besides respondent) ever spent the night in a cholera treatment unit?						
No	218	21	197	10.2 (6.06 - 15.7)	Ref	Ref
Yes	59	7	52	12.2 (4.82 - 23.6)	1.22 (0.45 - 3.32)	0.69
Observation questions						
Water storage vessel coverage (N = 243)						
Partially or not covered	27	1	26	4.24 (0.18 - 18.7)	Ref	Ref
Fully covered	216	23	193	10.6 (6.51 - 15.9)	2.67 (0.35 - 20.53)	0.34
Type of water storage vessel opening (N = 243)						
Narrow-mouthed	100	10	90	9.79 (4.54 - 17.6)	Ref	Ref
Wide-mouthed	143	14	129	10.3 (5.42 - 17.0)	1.05 (0.41 - 2.74)	0.91
Spigot or tap on water storage vessel (N = 243)						
No	166	18	148	10.9 (6.29 - 17.2)	Ref	Ref

Characteristics (N, if not 277)	Total (N = 277)	Unweighted Seropositive (N = 28)	Unweighted Seronegative (N = 249)	Weighted Seroprevalence % (95% CI)	Weighted Odds Ratio (95% CI)	p-value
Yes	77	6	71	7.31 (2.53 - 15.5)	0.64 (0.22 - 1.91)	0.42
Chlorine level (Parts per million[mg/L]) (N = 217)[e]						
0.1	152	16	136	9.38 (5.32 - 14.9)	Ref	Ref
0.2 - 2	43	2	41	9.05 (1.13 - 28.1)	0.96 (0.18 - 4.99)	0.96
2.1 - 6	22	2	20	10.2 (1.45 - 30.1)	1.09 (0.24 - 4.91)	0.91
Toilet type (N = 219)						
Open defecation	26	6	20	16.1 (4.52 - 35.8)	Ref	Ref
Unimproved	57	2	55	4.79 (0.85 - 13.8)	0.26 (0.04 - 1.53)	0.14
Improved	136	15	121	9.99 (5.55 - 16.0)	0.58 (0.16 - 2.03)	0.39

[a]Data are presented as the number (%) unless stated otherwise.
[b]These data are presented as the weighted median (Interquartile range). For this analysis, likelihood of poverty was calculated based on the $1.25 2005 purchasing power parity poverty line.
[c]We defined a household as an individual or group of related or unrelated individuals sleeping or staying under the same roof and sharing resources for at least half the week.
[d]HTG = Haitian Gourdes.
[e]Chlorine levels (based on chlorine free residual) of the household water supply was tested using a diethyl paraphenylene diamine (DPD) colorimetric indicator test (LaMotte, Chestertown, MD, USA).

References

1. Institut Haitiën de Statistiques et d'Informatiques. Population totale, population de 18 ans et plus, ménages et densités estimés en 2015. Port-au- Prince, Haiti: Ministère de l'Économie et des Finances; 2015 [cited 2023 Jan 5]. https://www.humanitarianresponse.info/sites/www.humanitarianresponse.info/files/documents/files/estimat_poptotal_18ans_menag2015.pdf

2. Schreiner M. Simple Poverty Scorecard poverty-assessment tool, Haiti [cited 2023 Feb 5]. https://www.simplepovertyscorecard.com/HTI_2012_ENG.pdf

3. Ballard T, Coates J, Swindale A, Deitchler M. Household hunger scale: indicator definition and measurement guide [cited 2023 Feb 5]. https://www.fantaproject.org/monitoring-and-evaluation/household-hunger-scale-hhs
